# Relationship between mean blood pressure during hospitalization and clinical outcome after acute ischemic stroke

**DOI:** 10.1186/s12883-023-03209-3

**Published:** 2023-04-20

**Authors:** Manyan Hu, Yuan Zhu, Zhaoyao Chen, Wenlei Li, Li Li, Yunze Li, Yangjingyi Xia, Tianrui Zhang, Qinghua Feng, Jiacheng Wu, Minghua Wu

**Affiliations:** Department of Neurology, Affiliated Hospital of Nanjing University of Chinese Medicine, Jiangsu Province Hospital of Chinese Medicine, 155 Hanzhong Road, Nanjing, 210029 China

**Keywords:** Acute ischemic stroke, Cerebral infarction, Blood pressure, Functional outcome

## Abstract

**Objective:**

The optimal blood pressure (BP) targets for acute ischemic stroke are unclear. We aimed to assess the relationship between Mean BP and clinical outcomes during hospitalization.

**Materials and methods:**

We included 649 patients with Acute ischemic stroke (AIS) from December 2020 to July 2021. BP was measured daily, and mean blood pressure was calculated. Clinical events recorded within 90 days of randomization were: recurrent ischemic stroke, symptomatic intracranial hemorrhage, and death. The modified Rankin Scale (mRS) was used to measure primary outcomes 3 months after AIS. Logistic multiple regression analysis was performed by statistical software R.

**Result:**

There is a nonlinear U-shaped relationship between SBP and poor outcomes. This means higher SBP and lower SBP will increase the incidence of poor outcomes. The optimal mean SBP during hospitalization was 135-150 mmHg, and patients with SBP < 135mmhg OR 2.4 [95% Cl, (1.16 ~ 4.97)], *P* = 0.018; and > 150mmhg OR 2.04 [95% Cl, 1.02 ~ 4.08], *p* = 0.045 had a higher probability of poor outcomes.

**Conclusion:**

Our study shows that the optimal SBP of patients with AIS during hospitalization was 135-150 mmHg. The findings suggest that the relationship between mean SBP and 3-month functional outcome after AIS was U-shaped. Both higher SBP and lower SBP lead to poor prognosis in AIS patients.

**Supplementary Information:**

The online version contains supplementary material available at 10.1186/s12883-023-03209-3.

## Introduction

Elevated blood pressure is common in the acute phase of stroke [1, 2], possibly as a compensatory mechanism to increase blood flow to the ischemic area [3]. BP control in the acute phase is an important factor affecting AIS patients’ clinical outcomes. However, it is generally believed that BP should not be excessively controlled in the acute stage of AIS as low BP may lead to ischemic tissue hypoperfusion [4, 5]. Poststroke hypotension is associated with different factors depending on stroke subtypes, such as cardioembolic stroke, which may be attributable to related heart failure [6], lacunar events attributable to coronary heart disease [6], and partial anterior circulation infarction attributable to previous myocardial infarction [7]. In addition, the stroke itself may cause damage to important vasomotor control centers (such as the hypothalamus and cortical vasomotor centers), particularly the right insular cortex, resulting in dysrhythmias, all potentially contributing to low BP [8]. Current guidelines about the management of poststroke hypotension provide no objective clarification on appropriate management, which is a reflection of the paucity of evidence in this field, and an indicator of the practical difficulties of carrying out research in the setting of acute stroke [9]. Therefore, some studies also support pressor therapy in response to acute hypotension [9]. However, increased BP during acute ischemic stroke may improve cerebral perfusion of ischemic tissue or may aggravate edema and hemorrhagic transformation of ischemic tissue [10]. Thus, it is important to raise blood pressure in an appropriate range. Both high and low blood pressure are independent predictors of poor outcomes. These associations appear to be partly mediated by higher rates of early recurrence and death in patients with high blood pressure and higher coronary heart disease events rates in those with low blood pressure [1].

At present, the blood pressure (BP) level that should be maintained in patients with AIS to ensure the best outcome is not known [11]. Therefore, ideal management in these situations should be individualized. Different subgroups of patients may need to have their BP lowered (e.g., before or after thrombolysis), left alone, or elevated [12].

Various studies have observed a U-shaped relationship between BP and clinical outcomes in many patients with ischemic stroke [1, 13–15]. Both extremes of BP were associated with poorer outcomes, although the reported optimal SBP varied widely from 120 to 130 mmHg [13, 14] to 156 to 220 mmHg in additional studies [16]. A J-shaped relationship between SBP and adverse outcomes has also been reported, whereas low SBP was not associated with functional outcomes [1, 17]. Because of these uncertainties, BP management during the acute stage of ischemic stroke remains an unresolved and controversial issue. The optimal blood pressure level to maintain in patients with AIS is unknown [11, 18]. Few studies have reported taking the mean blood pressure of AIS patients during hospitalization as the research object and comparing other BP values. The mean BP summarizes the overall level of BP of AIS patients during admission, which is an objective and stable value. We aimed to assess the relationship between mean BP and admission BP and clinical outcomes in patients with acute ischemic stroke.

## Methods

We used data from the stroke center of Jiangsu Province Hospital of Chinese medicine from December 2020 to July 2021. An observational cohort study of 649 patients with ischemic stroke was conducted. Patients were enrolled if they (1) had AIS and were seen within 72 h of symptom onset; (2) had acute ischemic lesions on the brain imaging; (3) had BP measurements q4 hours in the first 24 hours and then q8 hours after that. Patients with terminal malignant tumors or other underlying diseases like severe renal disease and liver disease, who failed to follow up at 90 days after the onset of symptoms, who had an mRS score ≥ 3 before onset, or who were treated with reperfusion, got excluded.

Potential risk factors for stroke, including hypertension (BP ≥ 140/90 mmHg, or previously used antihypertensive drugs), diabetes mellitus (previously used hypoglycemic drugs or glycated hemoglobin > 6.5% at admission), dyslipidemia (previously used lipid-lowering drugs or LDL ≥ 4.1 mmol/L, triglyceride ≥2.3 mmol/lL), coronary artery disease, smoking history, etc., were assessed.

## Measurement and management of BP

**Fig. 1:**
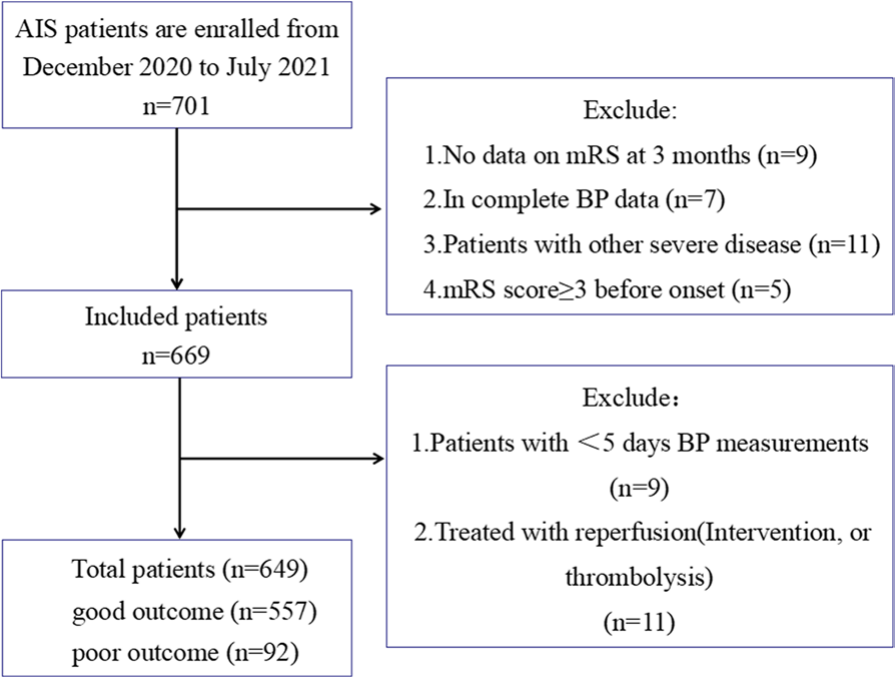
Flow Diagram

BP measurements were q4 h in the first 24 h and q8 h after that. Therefore, the mean value was calculated. BP was managed according to the Japanese Guidelines for Stroke Management [19, 20]. The mean blood pressure during the first 4 days of admission and from the fourth day of admission to discharge were evaluated. Data from BP measurements≥5 days were used to calculate the mean value in groups. After excluding patients hospitalized for less than 5 days, 649 patients were analyzed (Fig. 1).

### Clinical assessment and outcome measurements

The severity of neurological deficit was assessed during admission using the National Institutes of Health Stroke Scale (NIHSS) score [21]. The primary outcome was measured using the modified Rankin Scale (mRS) 3 months after the onset of symptoms. Clinical information of the results after discharge was obtained through follow-up 3 months after the qualifying event. An mRS score of 0-2 was defined as a favorable outcome (functional independence). Scores≥3 were considered poor outcomes. Moreover, clinical events were recorded, such as recurrent ischemic stroke, symptomatic intracranial hemorrhage, and death [1, 22, 23].

Brain imaging examination (CT/MRI) was performed during admission. According to the Trial of Org 10,172 in Acute Stroke Treatment classification, clinical and neuroimaging findings were used to classify patients into five-stroke subtypes: (1) large-artery atherosclerosis, (2) small-vessel occlusion, (3) cardioembolism, (4) stroke of other determined etiology, and (5) stroke of undetermined etiology [24].

### Statistical analysis

This study included 649 patients with AIS. BP was measured and entered into the database for all patients in the trial. Data are expressed as mean ± SD, IQR. Logistic regression analysis was used to determine the relationship between BP parameters (mean ± SD, IQR of SBP or DBP) and poor functional outcome.

The relationship between baseline BP and the functional prognosis was best fitted by smooth curve fitting, and reverse stepwise logistic regression analysis was used to evaluate the odds ratios (OR) and corresponding 95% confidence intervals (CIs) of the main results. BP was considered a continuous variable, and the optimal BP was determined by the dichotomous method based on threshold estimations for the main analysis. In addition, logistic regression analysis was performed to assess the optimal BP range [18]. All the analyses were performed using the statistical software R (http://www.R-project.org, The R Foundation) and Free Statistics software version 1.4 [25].

The primary outcome was the modified Rankin Scale (mRS) score at 90 days [26]. The mRS measures functional outcomes after stroke, ranging from 0 (no symptoms) to 6 (death). Scores ≥3 were defined as poor outcomes.

According to their clinical significance and the results of previous studies, the following variables were adjusted: age, sex, dyslipidemia, hypertension, diabetes mellitus, antihypertensive drugs, coronary heart disease, atrial fibrillation, and smoking. *P*-value < 0.05 was determined as the level of significance.

## Result

### Study population

Among 649 AIS patients during the study period, the median age was 69 (IQR 60-77) years, and 427 (65.8%) were male. In addition, 92 (14.18%) patients had poor outcomes, and 557 (85.82%) patients had good outcomes. mRS on admission was 2.0 (interquartile range, 1.0-3.0). Patients with poor outcomes were older than those with good outcomes. For patients with poor outcomes, more males than females, and more frequently have a medical history of diabetes or hypertension or use antihypertensive medication (Table 1).

**Table 1:** Clinical and biochemical characteristics of the good and poor outcomes in all AIS patients

Variables		Total (*n* = 649)	Good outcome (*n* = 557)	Poor outcome (*n* = 92)	*P*
Age, Median (IQR)		69.0 (60.0, 77.0)	67.0 (59.0, 76.0)	76.0 (68.8, 84.0)	< 0.001
Hospital stay (days), Mean ± SD		13.3 ± 4.1	13.0 ± 3.6	14.8 ± 6.1	< 0.001
Sex, n (%)	Male	427 (65.8)	378 (67.9)	49 (53.3)	0.009
	Female	222 (34.2)	179 (32.1)	43 (46.7)	
Risk factors, n (%)					
Hypertension	yes	490 (75.5)	417 (74.9)	73 (79.3)	0.426
	no	159 (24.5)	140 (25.1)	19 (20.7)	
Diabetes Mellitus	yes	272 (41.9)	219 (39.3)	53 (57.6)	0.001
	no	377 (58.1)	338 (60.7)	39 (42.4)	
Atrial Fibrillation	yes	73 (11.2)	51 (9.2)	22 (23.9)	< 0.001
	no	576 (88.8)	506 (90.8)	70 (76.1)	
Hyperlipidemia	yes	35 (5.4)	31 (5.6)	4 (4.3)	0.805
	no	613 (94.6)	525 (94.4)	88 (95.7)	
Coronary heart disease	yes	123 (19.0)	99 (17.8)	24 (26.1)	0.082
	no	526 (81.0)	458 (82.2)	68 (73.9)	
Smoking	yes	196 (30.2)	179 (32.1)	17 (18.5)	0.012
	no	453 (69.8)	378 (67.9)	75 (81.5)	
Drinking	yes	127 (19.6)	115 (20.6)	12 (13)	0.119
	no	522 (80.4)	442 (79.4)	80 (87)	
Biochemical variables, Median (IQR)					
Total-C		4.3 (3.4, 5.1)	4.3 (3.5, 5.1)	3.8 (3.0, 5.0)	0.012
TG		1.3 (1.0, 1.7)	1.3 (1.0, 1.7)	1.1 (0.9, 1.5)	0.005
HDL-C		1.2 (1.0, 1.4)	1.2 (1.0, 1.4)	1.2 (1.0, 1.5)	0.876
LDL-C		2.5 (1.9, 3.1)	2.6 (1.9, 3.2)	2.2 (1.6, 3.0)	0.011
TOAST classification, n (%)					< 0.001
LAA		301 (46.4)	250 (44.9)	51 (55.4)	
SVO		254 (39.1)	237 (42.5)	17 (18.5)	
CE		79 (12.2)	57 (10.2)	22 (23.9)	
OE and UD		15 (2.3)	13 (2.3)	2 (2.2)	
BP, n (%)/Mean ± SD					
Admission SBP		145.0 (132.0,162.0)	146.0 (133.0,161.0)	141.5 (126.5166.5)	0.782
Admission DBP		84.0 (75.0, 93.0)	84.0 (76.0, 92.0)	82.0 (73.8, 94.0)	0.363
Mean SBP		142.7 (133.0,151.8)	142.9 (133.7151.2)	141.6 (130.0,154.5)	0.736
Mean DBP, Mean ± SD		81.7 ± 9.0	82.1 ± 9.0	79.1 ± 9.1	0.002
BP indices in acute stage (1–4d), Mean ± SD					
Mean SBP		144.8 ± 16.4	144.6 ± 15.6	145.6 ± 20.6	0.618
Mean DBP		82.6 ± 9.7	82.9 ± 9.6	80.5 ± 10.3	0.027
BP indices in subacute stage (>4 d), n (%)/Mean ± SD					
Mean SBP		142.3 (132.2151.2)	142.4 (133.3151.1)	141.3 (128.5152.4)	0.563
Mean DBP		81.4 ± 9.3	81.9 ± 9.2	78.3 ± 9.3	< 0.001
Previous medication history, n (%)					
Antihypertensive drugs	yes	365 (56.2)	316 (56.7)	49 (53.3)	0.611
	no	284 (43.8)	241 (43.3)	43 (46.7)	
Hypoglycemic agents	yes	199 (30.7)	158 (28.4)	41 (44.6)	0.005
	no	449 (69.2)	398 (71.5)	51 (55.4)	
Statins	yes	97 (14.9)	78 (14)	19 (20.7)	0.134
	no	552 (85.1)	479 (86)	73 (79.3)	
Antiplatelet drugs	yes	120 (18.5)	95 (17.1)	25 (27.2)	0.03
	no	529 (81.5)	462 (82.9)	67 (72.8)	
Admission assessment, n (%)					
NIHSS		2.0 (1.0, 5.0)	2.0 (1.0, 4.0)	8.5 (5.0, 12.0)	< 0.001
mRS		2.0 (1.0, 3.0)	1.0 (1.0, 3.0)	4.0 (4.0, 5.0)	< 0.001
END	yes	90 (13.9)	64 (11.5)	26 (28.3)	< 0.001
	no	559 (86.1)	493 (88.5)	66 (71.7)	

*IQR* Interquartile range, *BP* indicates Blood pressure, *DBP* Diastolic blood pressure, *SBP* Systolic blood pressure, *TOAST* Trial of Org 10,172 in Acute Stroke Treatment, *LAA* Large-artery atherosclerosis, *SVO* Small-vessel occlusion, *CE* Cardioembolism, *OE and UD* stroke of other determined etiology and stroke of undetermined etiology, *TC* Total cholesterol, *TG* Triglyceride, *LDL* Low-density lipoprotein, *HDL* High-density lipoprotein, *AFib* Atrial fibrillation, *CHD* Coronary heart disease, *mRS* admission modified Rankin Scale, *END* Early neurological deterioration after stroke, *NIHSS* National Institutes of Health Stroke Scale

### BP grouping and clinical results

AIS patients with admission SBP and mean SBP and functional prognosis followed a ‘U-curve pattern’. Both extremes of BP were associated with poor outcomes. Although the correlation between SBP and the functional prognosis was significantly better than DBP, the correlation between DBP and prognosis was not statistically significant. The optimal range of mean blood pressure during hospitalization was between 135 mmHg and 150 mmHg.

**Fig. 2:**
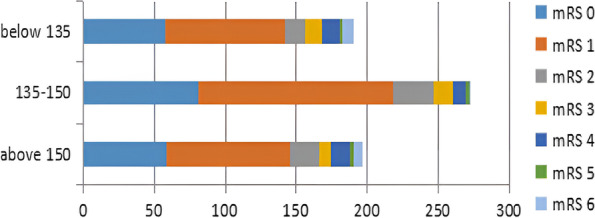
Distribution of scores on the modified Rankin Scale (mRS) at 90 d for patients with mean SBP: below 135 mmHg, between 135 and 150 mmHg and above 150 mmHg. x-axis: the Number of patients

Patients with mean SBP above 150 mmHg and below 135 mmHg had a higher median mRS score and more deaths at 90 days compared to patients with mean SBP between 135 and 150 mmHg (Fig. 2).

**Fig. 3:**
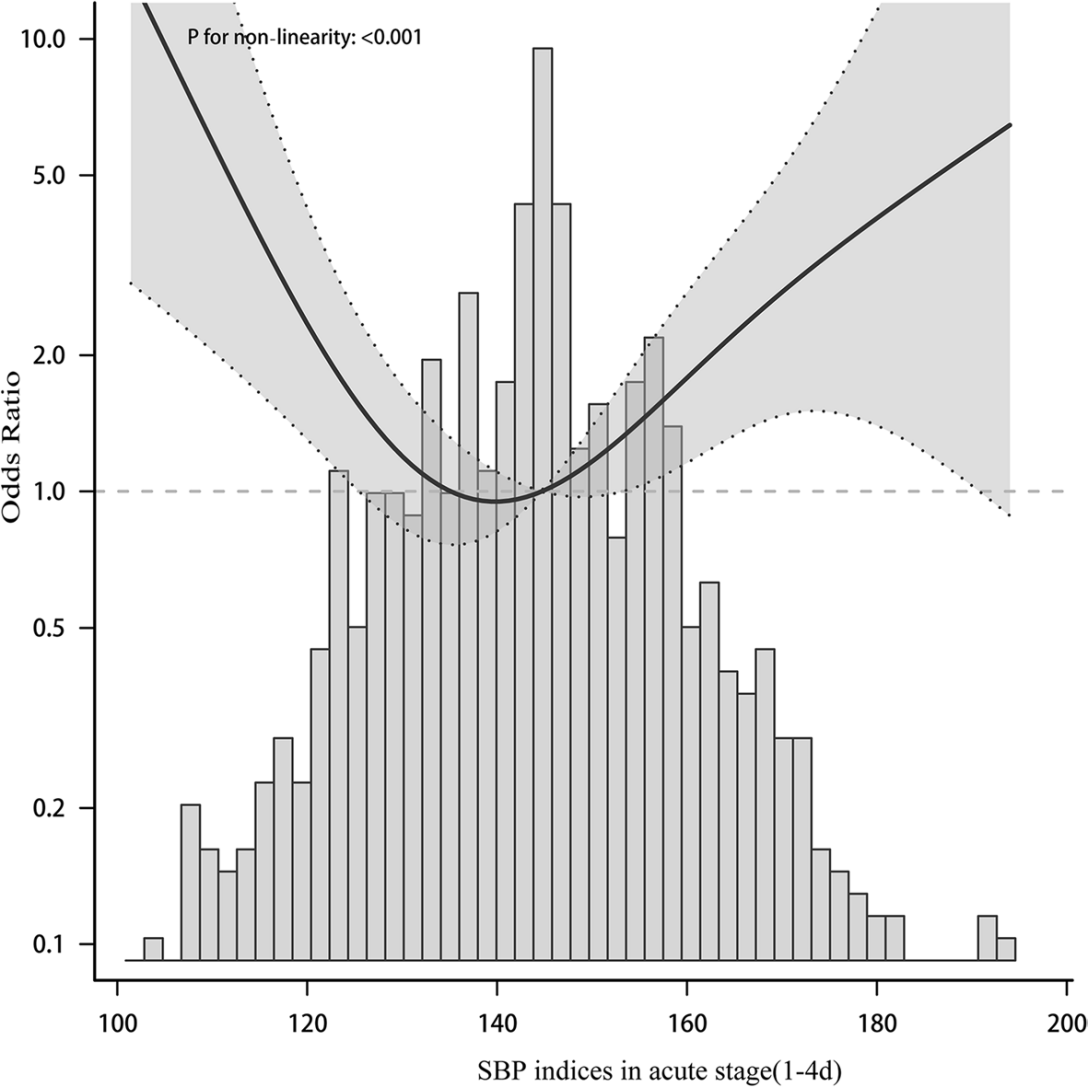
Relationship between acute phase SBP and functional outcome according to smooth fitting curve. Adjusting variables: TOAST, antihypertensive drugs, atrial fibrillation, coronary heart disease, hypertension, diabetes, hyperlipidemia, age, and gender

**Fig. 4:**
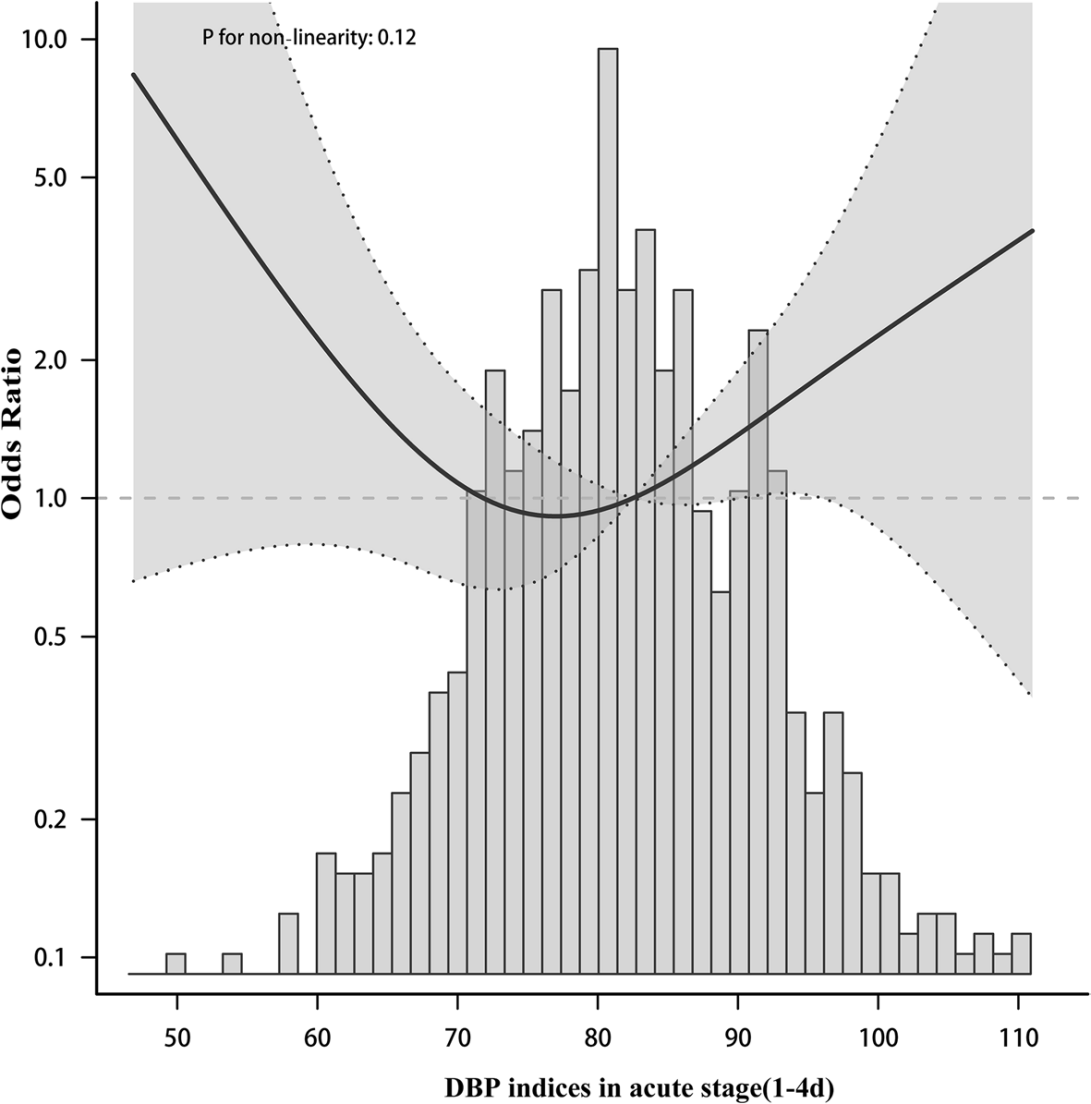
Relationship between acute phase DBP and functional outcome according to smooth fitting curve. Adjusting variables: TOAST, antihypertensive drugs, atrial fibrillation, coronary heart disease, hypertension, diabetes, hyperlipidemia, age, and gender

**Fig. 5:**
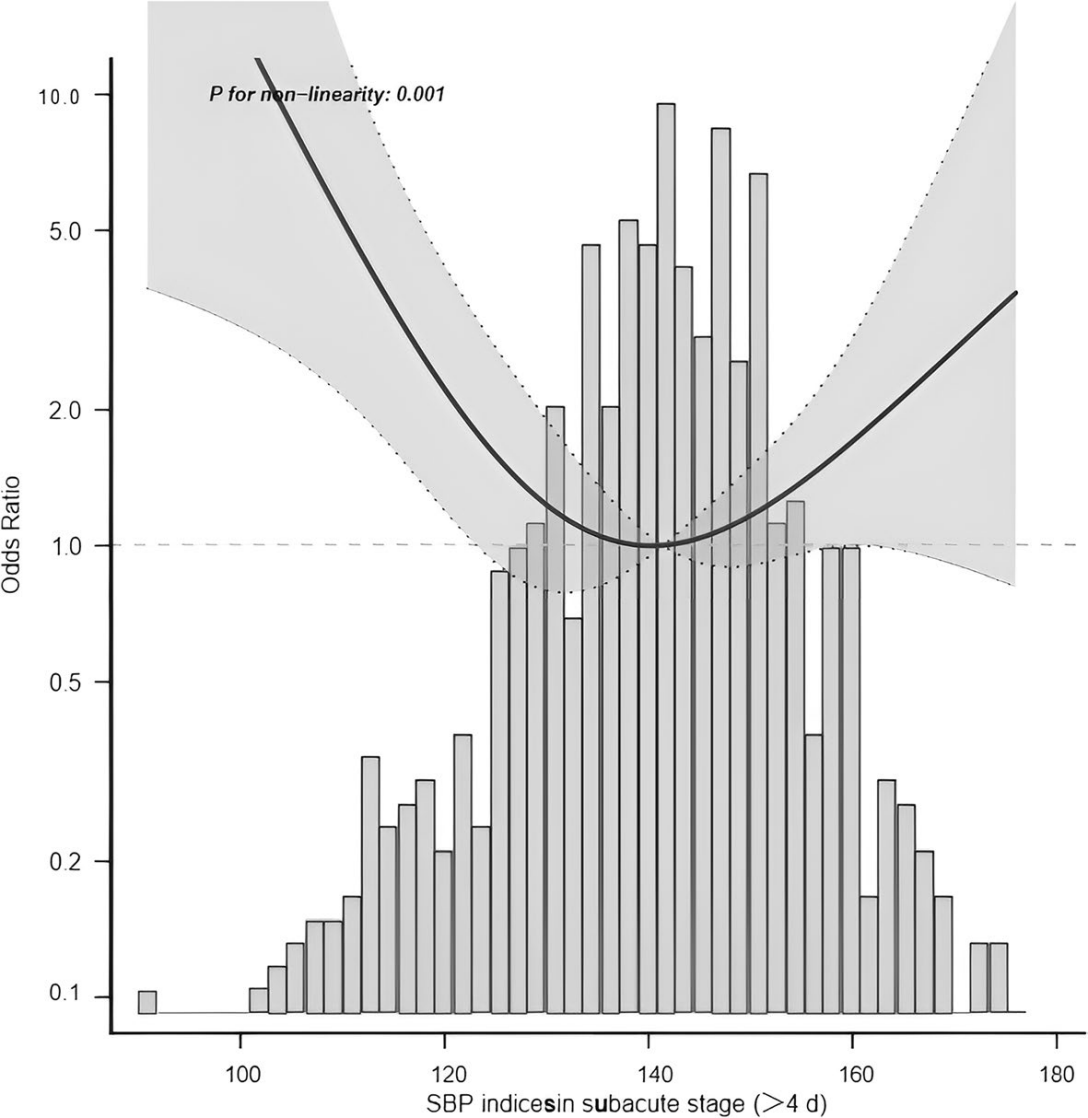
Relationship between subacute phase SBP and functional outcome according to smooth fitting curve. Adjusting variables: TOAST, antihypertensive drugs, atrial fibrillation, coronary heart disease, hypertension, diabetes, hyperlipidemia, age, and gender

**Fig. 6:**
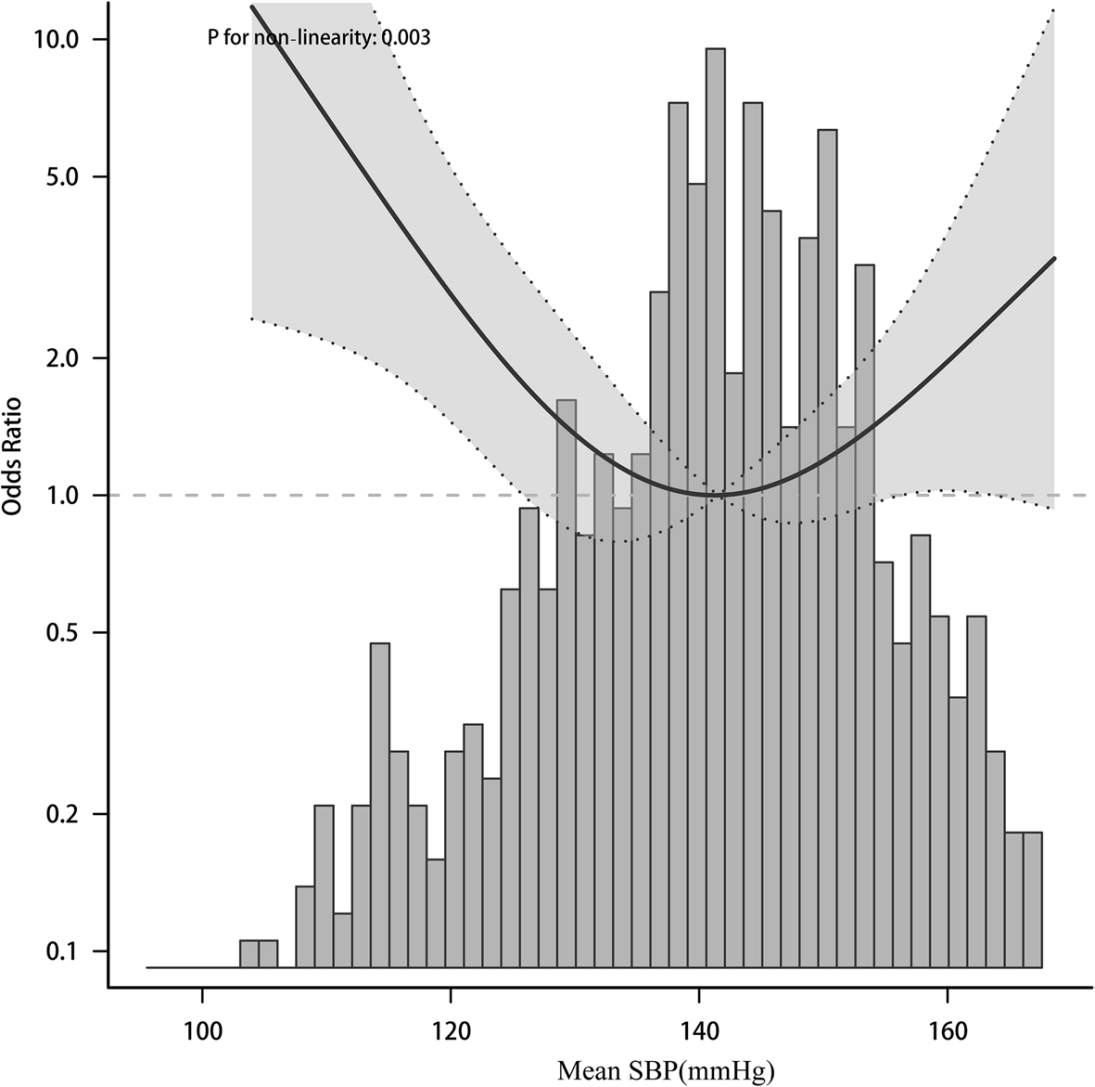
Relationship between subacute phase DBP and functional outcome according to smooth fitting curve. Adjusted variables: TOAST, antihypertensive drugs, atrial fibrillation, coronary heart disease, hypertension, diabetes, hyperlipidemia, age and gender

To elucidate whether mean BP during the acute stage days 1–4 after onset and days>4 was associated with functional outcome, Patients were divided into two groups according to daily BP. In the adjusted model, the nonlinear relationship between BP and functional results presented a symmetric U-shaped diagram (Figs. 3, 4, 5, 6). Day-by-day SBP during the acute 1-4 days and >4 days after onset was independently associated with a poor functional outcome, but DBP>4 days was not associated with a functional outcome. We evaluated according to the cut-points (135 mmHg) in the fitted curves (Fig. 3 and Table 2). Threshold effect analysis using piecewise linear regression (Table 2) showed that when BP was < 138 mmHg, the odds of outcomes negatively correlated with BP [OR 0.936 (0.882 ~ 0.992) *p* = 0.0258]. However, when the BP was > 135 mmHg, the incidence of poor outcomes increased with increasing SBP [OR 1.036 (1.008 ~ 1.066) *p* = 0.0125].

**Table 2:** Relationship between 1-4d SBP and functional outcome

1-4d SBP	OR (95%CI)	*P*
< 135	0.936 (0.882 ~ 0.992)	0.0258
> 135	1.036 (1.008 ~ 1.066)	0.0125

### Sensitivity analysis

**Fig. 7:**
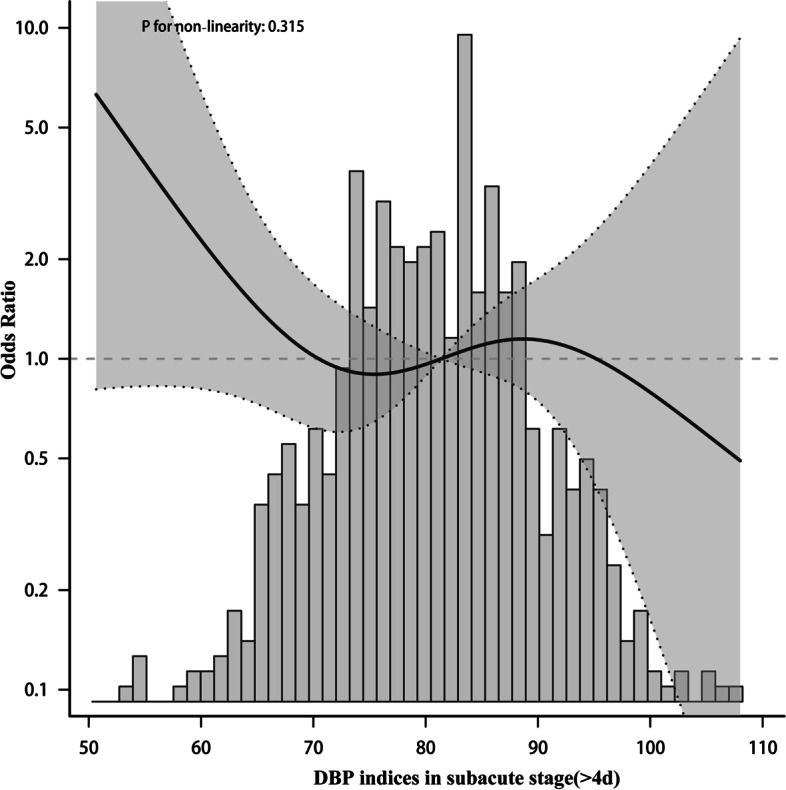
Relationship between mean SBP and functional outcome according to smooth fitting curve. Adjusting variables: TOAST, antihypertensive drugs, atrial fibrillation, coronary heart disease, hypertension, diabetes, hyperlipidemia, age, and gender

Both higher SBP and lower SBP lead to poor prognosis in AIS patients. In the following analysis, restricted cubic spline smoothing curve fitting was used to assess the relationship between BP and functional outcome, showing that SBP and functional results have a ‘U-curve pattern (Fig. 7).

We used piecewise linear regression analysis according to the best range of fitting results (Table 3). Further confirm the range of mean systolic blood pressure. The piecewise linear regression showed that 135-150 mmHg is the best blood pressure range that can be determined in this study during hospitalization. Lower and higher blood pressures were significantly associated with functional outcomes. BP < 135 mmHg(OR 1.93 [95%Cl, OR 1.11-3.36], *P* = 0.021; After adjustment, OR 2.4 [95%Cl, 1.16-4.97], *P* = 0.018). And BP > 150 mmHg(OR 1.95 [95%Cl,1.12-3.37], *P* = 0.017; Adjusted OR 2.04 [95%CL, 1.02-4.08], *P* = 0.045) (Table 3).

**Table 3:** Relationship between mean SBP and functional outcome indifferent models

Variable	n.total	n.event_%	crude.OR_95CI	crude.P_	adj.OR_95CI	adj.*P*_value
135-150	267.0	26 (9.7)	1(Ref)		1(Ref)	
< 135	186.0	32 (17.2)	1.93 (1.11 ~ 3.36)	0.021	2.4 (1.16 ~ 4.97)	0.018
> 150	196.0	34 (17.3)	1.95 (1.12 ~ 3.37)	0.017	2.04 (1.02 ~ 4.08)	0.045
Trend.test	649.0	92 (14.2)	1.01 (0.76 ~ 1.35)	0.932	0.95 (0.64 ~ 1.4)	0.792

Adjusted variables: TOAST, antihypertensive drugs, atrial fibrillation, coronary heart disease, hypertension, diabetes, hyperlipidemia, age and gender

**Fig. 8:**
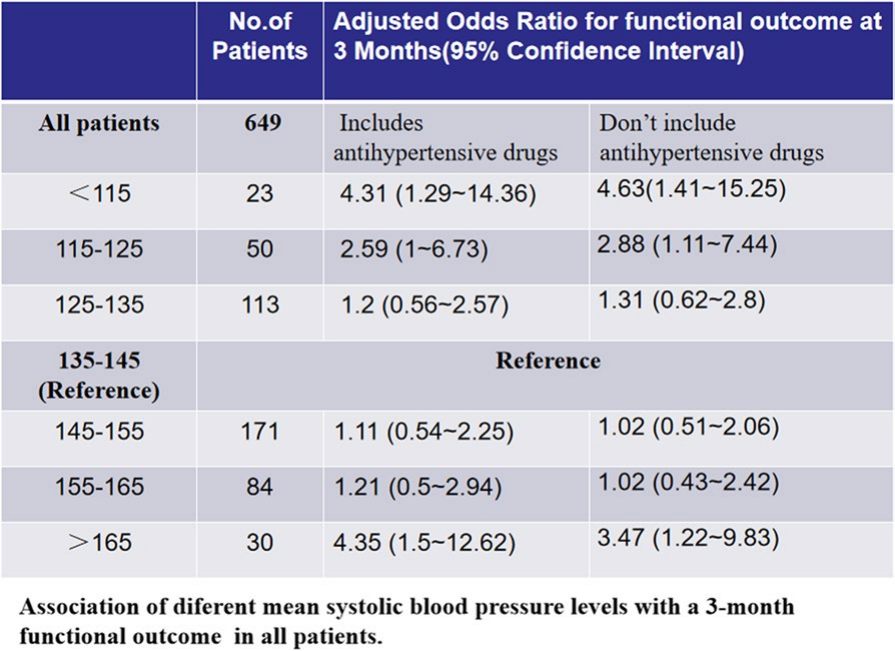
Association of different mean systolic blood pressure levels with a 3-month functional outcome in all patients. A multiple logistic regression test was used to analyze odds ratios. CI, confidence interval. Adjusted variables: TOAST, antihypertensive drugs, atrial fibrillation, coronary heart disease, hypertension, diabetes, hyperlipidemia, age and gender

Because there was significant discussion about antihypertensive therapy in the guidelines for post stroke therapy. We adjusted variable with and without antihypertensive therapy. we used logistic regression analysis to evaluate the blood pressure data at 10 mmHg interval with 135-145 mmHg as reference. The adjusted values showed the relationship between different blood pressure values and functional outcomes (Fig. 8), a significant increase in the probability of a poor functional outcome in patients with SBP < 135 mmHg or > 145 mmHg (vs. > 135 to ≤145 mmHg). The results indicate that either with or without antihypertensive treatment our conclusions are stable, lower and higher blood pressure are significantly associated with functional outcomes, which further confirmed our U-shaped curve.

## Discussion

This Retrospective study demonstrates that SBP is significantly correlated with the functional outcome of AIS. There was a nonlinear U-shaped relationship between the SBP level during hospitalization and the mRS scores at 3 months after AIS in these patients. Furthermore, comparing admission SBP, mean SBP during hospitalization correlated more with the functional outcome at 3 months after AIS in patients. Moreover, maintaining a mean SBP of 135-150 mmHg during hospitalization is associated with higher odds of a favorable outcome 3 months after AIS.

Some observational studies show an association between worse outcomes and lower BP, whereas others have not [1, 11, 14, 16]. Although in other reports, most studies targeted BP at admission or the mean BP measured within 24 or 72 h after the onset of symptoms [14–16], studies of overall mean BP during hospitalization are rare. Due to the patient’s personal reasons or other influencing factors, SBP at admission is difficult to control. Whether to take antihypertensive measures after admission also needs to consider various factors. We assume that the overall mean value of daily BP at a fixed time is more reliable as a long-term index than a random or a single BP value at a specific time point because it is less affected by various factors.

Taking the mean BP during hospitalization as the main research object is more stable and objective. Moreover, our study demonstrates that mean BP is more stable during hospitalization, and both low BP levels and high BP levels were associated with poor outcomes. Furthermore, SBP maintained between 135 and 150 may be optimal for AIS patients during hospitalization.

In the subgroups of this study, the mean BP in the acute (1-4 days after onset) and subacute (>4 days) stages were equally stable, and both were related to the functional outcome, but the difference was not significant.

In another study, BP during the subacute phase was significantly associated with functional outcomes after ischemic stroke, but there was no association between BP during the acute stage and functional outcome [27]. The results in the present study may be related to the widespread use of antihypertensive drugs in China. According to the latest guidelines, patients with AIS need emergency BP reduction to prevent serious complications [11]. However, excessive lowering of BP can sometimes aggravate cerebral ischemia [28]. Therefore, ideal BP management should be personalized. In this study, antihypertensive drugs were not classified but were adjusted in statistical analysis. The impact of antihypertensive drug use on outcomes has been reflected in the adjusted model.

The change in BP is affected by many factors, including increased sympathetic drive, reduced arterial and cardiopulmonary reflexes, impaired arterial compliance, humoral factors, blood viscosity, and emotional factors [29, 30]. Sykora et al. [31] hypothesized that autonomic dysfunction in AIS patients might have unfavorable effects on outcomes through secondary brain injury caused by cerebral hypoperfusion, impaired brain autoregulation, and cardiovascular complications. These conditions may exacerbate cerebral ischemia or hinder recovery, leading to poor functional outcomes after AIS [27]. Further studies are needed to elucidate the association mechanism between blood pressure changes and post-stroke functional outcomes.

A potential limitation of this study is a single-center study with a small number of samples, which may lead to an underestimation of the true association. Furthermore, the study is retrospective, and the recorded data may be incomplete. Patients with short hospital stays and fewer blood pressure measurements were excluded, resulting in potential selection bias. Furthermore, unidentified confounders may not be adjusted, and the data must be interpreted cautiously. Further studies are required to elucidate the mechanism and clinical significance of the relationship between mean blood pressure and functional outcome in patients with acute ischemic stroke during hospitalization.

## Conclusion

The result of the present study indicates that maintenance of mean BP between 135 mmHg and 150 mmHg in AIS patients during hospitalization is associated with promising short- or long-term clinical outcomes. These findings have significant potential implications for managing BP in acute stroke, as they support the need to initiate treatment for high BP early rather than conservatively delaying therapy to a certain point after symptom onset. The overall BP stability has a more objective value than the fixed time.

## Supplementary Information

**Additional file 1: Table S1.** Relationship Between >4d SBP and functional outcome. **Table S2.** Relationship Between mean SBP and functional outcome. **Table S3.** Relationship Between admission SBP and functional outcome. **Table S4.** Relationship between admission SBP and functional outcome indifferent models. **Fig. S1.** Relationship between mean DBP and functional outcome according to smooth fitting curve. Adjusting variables: TOAST, antihypertensive drugs, atrial fibrillation, coronary heart disease, hypertension, diabetes, hyperlipidemia, age, and gender. **Fig. S2.** Relationship between Admission SBP and functional outcome according to smooth fitting curve. Adjusting variables: TOAST, antihypertensive drugs, atrial fibrillation, coronary heart disease, hypertension, diabetes, hyperlipidemia, age, and gender.

## Data Availability

All data are available from the corresponding author upon reasonable request.
