# Supplementary material for: Relationship between mean blood pressure during hospitalization and clinical outcome after acute ischemic stroke
Source: BMC Neurol. 2023 Apr 20;23:156. doi: 10.1186/s12883-023-03209-3 (PMC10116692; doi:10.1186/s12883-023-03209-3)
Supplement: Supplementary file 1 — Additional file 1: Table S1. Relationship Between >4d SBP and functional outcome. Table S2. Relationship Between mean SBP and functional outcome. Table S3. Relationship Between admission SBP and functional outcome. Table S4. Relationship between admission SBP and functional outcome indifferent models. Fig. S1. Relationship between mean DBP and functional outcome according to smooth fitting curve. Adjusting variables: TOAST, antihypertensive drugs, atrial fibrillation, coronary heart disease, hypertension, diabetes, hyperlipidemia, age, and gender. Fig. S2. Relationship between Admission SBP and functional outcome according to smooth fitting curve. Adjusting variables: TOAST, antihypertensive drugs, atrial fibrillation, coronary heart disease, hypertension, diabetes, hyperlipidemia, age, and gender. [file 12883_2023_3209_MOESM1_ESM.docx]

**Table S1.** Relationship Between ＞4d SBP and functional outcome

| ＞4d SBP | OR（95%CI） | P |
| --- | --- | --- |
| <134 | 0.944 (0.896~0.995) | 0.0316 |
| >134 | 1.034 (1~1.068) | 0.0492 |

| Mean SBP | OR（95%CI） | P |
| --- | --- | --- |
| <138 | 0.95 (0.905~0.998) | 0.0427 |
| >138 | 1.035 (0.997~1.074) | 0.0683 |

**Table S2.** Relationship Between mean SBP and functional outcome

**Table S3.** Relationship Between admission SBP and functional outcome

| Admission SBP | OR（95%CI） | P |
| --- | --- | --- |
| <148 | 0.971 (0.948~0.996) | 0.023 |
| >148 | 1.028 (1.005~1.052) | 0.0185 |

**Table S4.** Relationship between admission SBP and functional outcome indifferent models

Variable n.total n.event_% crude.OR_95CI crude.P_ adj.OR_95CI adj.P_value

145-160 152.0 10 (6.6) 1(Ref) 1(Ref)

<145 309.0 48 (15.5) 2.61 (1.28~5.32) 0.008 2.75 (1.17~6.45) 0.02

>160 188.0 34 (18.1) 3.14 (1.49~6.58) 0.003 2.28 (0.93~5.59) 0.073

Trend.test 649.0 92 (14.2) 1.06 (0.82~1.36) 0.678 0.88 (0.63~1.22) 0.433

Adjusted variables: TOAST, antihypertensive drugs, atrial fibrillation, coronary heart disease, hypertension, diabetes, hyperlipidemia, age and gender


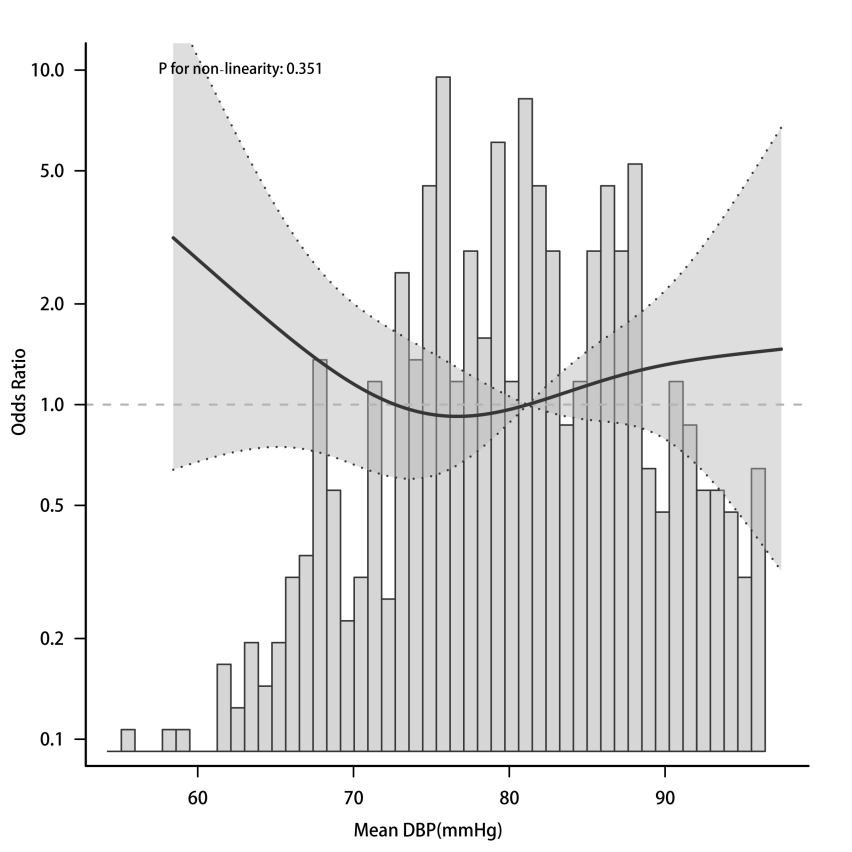


**Figure S1.** Relationship between mean DBP and functional outcome according to smooth fitting curve. Adjusting variables: TOAST, antihypertensive drugs, atrial fibrillation, coronary heart disease, hypertension, diabetes, hyperlipidemia, age, and gender.


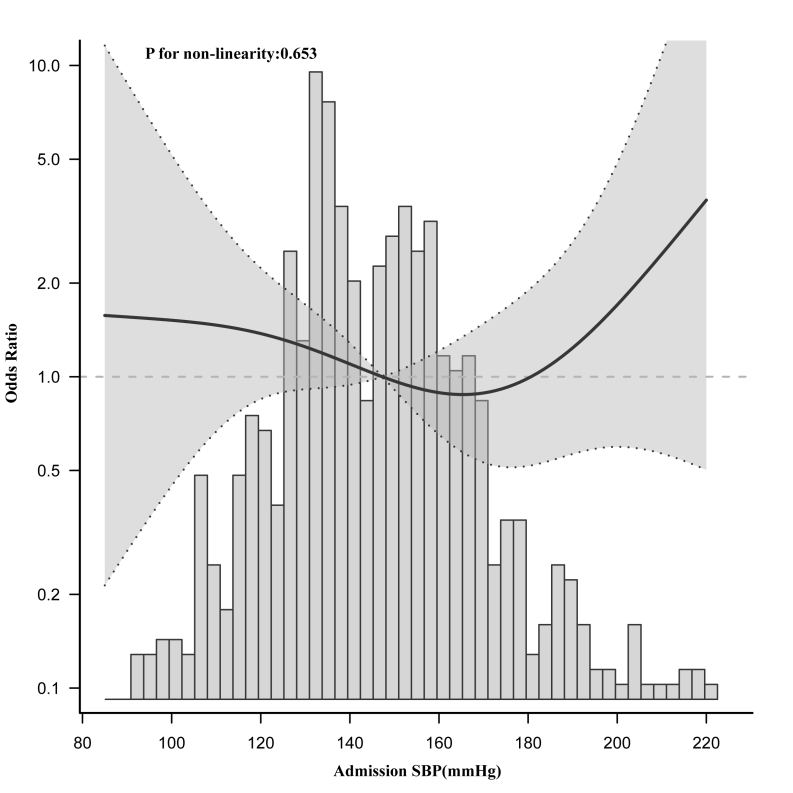


**Figure S2.** Relationship between Admission SBP and functional outcome according to smooth fitting curve. Adjusting variables: TOAST, antihypertensive drugs, atrial fibrillation, coronary heart disease, hypertension, diabetes, hyperlipidemia, age, and gender.
